# The Effect of Using New Synbiotics on the Turkey Performance, the Intestinal Microbiota and the Fecal Enzymes Activity in Turkeys Fed Ochratoxin A Contaminated Feed

**DOI:** 10.3390/toxins12090578

**Published:** 2020-09-09

**Authors:** Katarzyna Śliżewska, Paulina Markowiak-Kopeć, Anna Sip, Krzysztof Lipiński, Magdalena Mazur-Kuśnirek

**Affiliations:** 1 Institute of Fermentation Technology and Microbiology, Department of Biotechnology and Food Sciences, Lodz University of Technology, Wólczańska 171/173, 90-924 Łódź, Poland; 2 Department of Biotechnology and Food Microbiology, Poznan University of Life Sciences, Wojska Polskiego 48, 60-627 Poznań, Poland; anna.sip@up.poznan.pl; 3 Department of Animal Nutrition and Feed Science, University of Warmia and Mazury, Oczapowskiego 5/248, 10-719 Olsztyn, Poland; krzysztof.lipinski@uwm.edu.pl (K.L.); magdalena.mazur@uwm.edu.pl (M.M.-K.)

**Keywords:** synbiotics, turkeys, intestinal microbiota, fecal enzymes, ochratoxin A

## Abstract

The feed supplementation of probiotic microorganisms is a promising method for detoxification of ochratoxin A (OTA) in poultry. The aim of the study was to investigate the effect of newly elaborated synbiotics on the turkey performance, the intestinal microbiota and its enzymatic activity in turkeys (0–15 weeks) fed OTA contaminated feed (198.6–462.0 µg/kg) compared to control group (OTA-free feed). The studies determined the composition of intestinal microorganisms by the culture method and the activity of fecal enzymes by spectrophotometry. It was found that OTA had an adverse effect on the body weight, the intestinal microbiota and the fecal enzymes activity in turkeys. On the other hand, synbiotics resulted in an increase in the count of beneficial bacteria while reducing the number of potential pathogens in the digestive tract. Moreover, synbiotics caused an increase in the activity of α-glucosidase and α-galactosidase, while decreasing the activity of potentially harmful fecal enzymes (β-glucosidase, β-galactosidase, β-glucuronidase) in the turkey’s excreta. Results indicate a beneficial effect of elaborated synbiotics on the health of turkeys and a reduction of the negative impact of OTA contaminated feed. These synbiotics can be successfully used as feed additives for turkeys.

## 1. Introduction

The task of rational nutrition of farm animals is not only to obtain the maximum production result but also to care for animal welfare through a beneficial effect on the digestive tract, metabolism and stimulation of the immune system. An important factor responsible for the deterioration of production results, damage to the liver and other organs and the weakening of the immune system are mycotoxins [1,2]. Over 500 mycotoxins are known, and only about 50 are relatively well characterized [3]. The best feed components are used in mixtures for poultry, rarely containing an increased number of mycotoxins. However, the results of many studies indicate that even relatively low levels of mycotoxins in feed for young animals can cause a weakening of the immune system or reduce feed intake, and an increased content of mycotoxins in feed, especially containing corn grain and wheat bran, occurs in practice [4]. It is assumed that about 25% of the world’s harvest may be contaminated with mycotoxins [3]. Mycotoxins are dangerous, and low doses lead to a weakening of the immune system and a decrease in animal performance [1,5,6]. Aflatoxins (AFB1, AFM1) and ochratoxin A (OTA) are commonly present in poultry feeds. Losses in poultry farming caused by these substances are mainly due to a decrease in production results and slaughter efficiency. In addition, there is a reduction in the quality of animal products and increased immunosuppression [3,7]. Penetration of mycotoxins is possible through the respiratory and digestive system of birds, and their metabolites accumulating in meat and eggs can negatively affect human health [2]. OTA belongs to the carcinogenic mycotoxins and can be produced by many species of *Aspergillus* or *Penicillium*. The European Commission Recommendation 2006/576/EC suggests that the maximum level of OTA in poultry feeds should be set to 0.1mg OTA kg^−1^ [8]. One of the effects of its action on the digestive system is damage to the intestinal mucosa. Damage to the intestinal barrier, epithelial necrosis, impaired glucose absorption and intense diarrhea expose animals to colonization by harmful microorganisms, e.g., *Salmonella* Typhimurium [9]. Adverse effects of OTA in poultry are manifested by increased mortality, limited feed intake and nephrotoxicity [10]. The negative effects of OTA on performance traits of poultry have been confirmed by many authors [11,12,13,14]. Symptoms of poisoning poultry due to the presence of OTA may include weight loss, depression and muscle paralysis. In addition, there is a decrease in mobility and energy in poultry, as well as rapid breathing; catarrhal inflammation of the gastrointestinal mucosa; focal hyperemia and especially local ecchymoses on the surface of the liver, kidneys and less often the heart [15].

The use of feed additives such as probiotics, prebiotics and synbiotics is beneficial for the balance of the intestinal microbiota of poultry and may minimize the adverse effects of mycotoxins to which the animals are exposed [16,17,18]. According to the definition formulated in 2002 by World Health Organization (WHO) and Food and Agriculture Organization of the United Nations (FAO), probiotics are “live microorganisms which, when administered in sufficient amounts, confer a health benefit on the host” [19]. This definition was maintained in 2013 by the International Scientific Association for Probiotics and Prebiotics (ISAPP) and is still presently used [20]. In other side, prebiotics are defined as “a nonviable food component that confers a health benefit on the host associated with modulation of the microbiota” [21]. Finally, synbiotics are formulas containing both synergistically acting prebiotics and probiotics. Detoxification by probiotic microorganisms may occur through adsorption or as a result of microbial metabolism of the mycotoxin [17,18]. Not all microorganisms have detoxifying properties, these properties are mainly related to the strain and not the species. Microorganisms with confirmed detoxification properties are *Acinetobacter calcoaceticus*, *Bifidobacterium* spp. and *Enterococcus faecium* [22,23]. However, particular interest should be paid to *Saccharomyces cerevisiae* yeast and lactic acid bacteria (LAB) strains. Scientific evidence shows that detoxification by lactic acid bacteria and yeast is the result of adhesion to cell wall structures, confirming the fact that even dead cells retain this activity [24].

The beneficial effect of the new elaborated synbiotic preparations on the intestinal microbiota of poultry has so far been confirmed in studies on SPF (specific pathogen free) and broiler chickens [25,26]. In chickens, the effect of these synbiotics on dominant intestinal microorganisms was tested, and it was found that synbiotic preparations increase the number of beneficial microorganisms such as *Bifidobacterium* spp. and *Lactobacillus* spp., while reducing the number of potentially pathogenic bacteria such as *Clostridium* spp. and *Escherichia coli*. In addition, promising results have been obtained in in vitro as well as in vivo studies on OTA detoxification [16]. Due to the beneficial effect of synbiotics on the health of chickens, we also decided to examine the effect in turkeys.

Confirmation of the effectiveness of the developed synbiotics in the feeding of turkeys is an important area of research feed additives that can potentially replace antibiotic growth promoters. The aim of the research was to evaluate of effects newly elaborated synbiotic preparations on the turkey performance, the intestinal microbiota and activity of fecal enzymes in turkeys Big-6 fed ochratoxin A contaminated feed. The analyzes were carried out for animals fed with the administration of synbiotic preparations (A, B or C) and commercial probiotic preparations (BioPlus 2B or Cylactin) and also comparative control animals (no feed additives).

## 2. Results and Discussion

### 2.1. The Effect of Tested Formulas on Turkey Performance

Determining the performance of farm animals is very important in testing the effectiveness of feed additives such as probiotics, prebiotics or synbiotics. It was found that after 6 weeks the average body weight of turkeys was significantly higher (by approx. 8%) for turkeys consuming feed supplemented with synbiotic A and C in comparison to OTA group and turkeys consuming the feed with probiotics (BioPlus 2B, Cylactin) (Table 1). After 15 weeks, the highest weight of animals was found in the control group receiving OTA-free feed and was higher by an average of 2 kg compared to animals receiving OTA-contaminated feed without additives. FCR after 15 weeks of bird’s life was lower for turkeys consuming feed with synbiotic A compared to other groups consuming feed supplemented synbiotics or probiotics, but the observed differences were not statistically significant. The OTA contamination of feed did not significantly affect the mortality rate of the turkeys. After 6 weeks, EPEF was significantly higher in the control group (248.18) and in groups of turkeys consuming feed supplemented with synbiotic A (189.60) and synbiotic C (182.02) in comparison to OTA group (169.66) and animals consuming feed with probiotics (165.06 and 165.17). On the other hand, after 15 weeks, EPEF was the highest in the control group of animals (393.65) (Table 1).

The addition of probiotic, prebiotic and synbiotic preparations to poultry feed mixtures can reduce the negative effects of mycotoxins, which has been confirmed in many studies [16,27,28,29,30]. The ability of probiotic microorganisms (e.g., LAB, *S. cerevisiae*) to detoxify OTA leads to stabilization of the intestinal microbiota and improvement of rearing rates [17,18,23]. Santin et al. have shown that the use of *S. cerevisiae* cell walls under exposure to OTA in compound feed for broiler chickens can improve FCR [31]. The authors found no effect of the additive on other growth parameters. On the other hand, the use of esterified glucomannan in compound feed contaminated with OTA and other mycotoxins has increased the weight gain of broiler chickens [11]. The use of synbiotic preparations tested in this research improved the body weight of chickens exposed to OTA [16]. However, in this study, feed supplementation with synbiotics caused a slight increase in body weight of turkeys; however, it was statistically significantly lower compared to the control group.

### 2.2. The Effects of Synbiotics on the Ochratoxin A Content in Tissues and Intestinal Contents of Turkeys

Turkeys consuming feed supplemented with probiotics and synbiotics were characterized by lower OTA concentrations in the liver relative to birds from OTA group after 6 and 15 weeks of life (Table 2). However, it was only after 15 weeks that the differences were statistically significant. Synbiotic A and synbiotic B caused the greatest reduction in the OTA concentration in the liver after 15 weeks.

After 6 weeks of experiment, the lowest OTA concentration was noted in the kidneys of turkeys consuming feed supplemented with synbiotic A in comparison to birds from control-positive group (Table 2). Dietary supplementation with synbiotics (A, B and C) decreased the content of OTA in kidneys of turkeys in 15 weeks of experiment compared to control-positive group and birds consuming feed with Cylactin and BioPlus 2B.

After 6 weeks of birds’ life, the concentration of OTA in the jejunum content of turkeys consuming feed supplemented with synbiotic A (57.04 µg/kg) was the lowest (Table 2). Moreover, the concentration of OTA in the jejunum content in animals fed with feed with the addition of synbiotic B and C (150.97 and 106.83 µg/kg, respectively), and also BioPlus 2B (102.81µg/kg) and Cylactin (126.07 µg/kg) was lower compared to control-positive group. After 15 weeks of experiment, the supplementation of synbiotic A and B was the most effective, and the concentration of OTA was, respectively, 97.40 and 96.21 µg/kg (Table 2).

Turkeys consuming feed supplemented with synbiotics A, B and probiotic BioPlus 2B had lower OTA concentration in the content of caecum (78.79, 46.87 and 53.73 µg/kg, respectively) in comparison to other groups of animals after 6 weeks rearing (Table 2). After 15 weeks of experiment, the concentration of OTA in the caecum content of birds consuming feed supplemented with synbiotics A, B and C (34.63, 54.93, 45.98 µg/kg respectively) was significantly lower in comparison to control-positive group of turkeys (Table 2).

The main site of OTA absorption is the small intestine, especially the proximal jejunum. Absorption occurs through both active transport and passive diffusion. After being absorbed with the blood of the portal vein, mycotoxins enter the liver and other organs [32]. Subsequently, mycotoxins can be partially metabolized in the body, excreted in urine, bile and feces, or accumulated in the liver, kidneys and muscles [32].

In the study of Pozzo et al., OTA residues were detected in kidneys (3.58 ng/g) and liver (1.92 ng/g) of broiler chickens receiving OTA contaminated mixtures in the amount of 0.1 mg/kg [33]. Similar results were obtained in other studies [34,35]. Zaghini et al. found that esterified glucomannan and *Saccharomyces cerevisiae* do not reduce the concentration of mycotoxins in kidneys of chickens fed with OTA contaminated feed at 2 mg/kg [36]. The administration of synbiotics tested in this research caused a decrease in concentration of OTA and the genotoxicity of fecal water in broiler chickens contaminated OTA [16]. On the other hand, Kozaczyński found that broiler chickens fed OTA contaminated mixtures (0.2 mg/kg) for 20 weeks did not cause OTA accumulation in the kidneys or liver [37]. Differences in OTA concentrations between organs result from the length of exposure and the concentration of mycotoxins in bird’s feed doses [38]. The particularly strong affinity of OTA for accumulation in the liver and kidneys may be due to the function of these organs responsible for detoxification and excretion of toxins [39,40]. The use of probiotics, prebiotics or synbiotics can be a solution that limits the negative impact of OTA on monogastric animals. Preparations of this type can bind mycotoxins in the gastrointestinal tract (GIT), thus reducing their absorption and systemic toxicity, which was confirmed by our own research [24,41].

### 2.3. The Effect of Synbiotics on the Dominant Intestinal Microbiota of Turkeys

In the study, contents of the jejunum and the caecum were examined after 6 and 15 weeks, while the excreta were examined after 3, 9 and 15 weeks of animal rearing. The number of anaerobic bacteria in the content of the jejunum, the caecum and the excreta did not change significantly during 15 weeks of turkeys rearing (Table S1). This is a beneficial effect, as the balance of intestinal microbiota in turkeys is maintained. The number of anaerobic bacteria in the excreta was similar in each group after 3 weeks. In a further period of turkeys rearing, no differences were statistically significant between groups of birds, and after 6 weeks, the numbers of anaerobic bacteria were on average 1.95 × 10^8^–1.37 × 10^9^ CFU/g (the jejunum) and 1.25–6.10 × 10^9^ CFU/g (the caecum). After 15 weeks of rearing, it was found that the total numbers of anaerobic bacteria were, respectively, 2.60 × 10^8^–2.97 × 10^9^ CFU/g (the jejunum), 1.05–5.67 × 10^9^ CFU/g (the caecum) and 6.12 × 10^8^–2.19 × 10^9^ CFU/g (the excreta) (Table S1). These results were higher on average by one order of magnitude in comparison to the results reported by Dibaji, Seidavi, Asadpour and da Silva, who tested the effect of the Biomin IMBO synbiotic, containing the probiotic bacteria *Enterococcus faecium* (5 × 10^11^ CFU/kg) and fructooligosaccharides (a prebiotic), administered for 42 days, on the intestinal microbiota of chickens [42].

During the experiment, in samples of the intestinal content and the excreta, the average number of *Enterobacteriaceae* family bacteria was similar in turkeys consuming fed supplemented with probiotics, synbiotics and also control groups (positive and negative) of animals. After 15 weeks of bird’s life, the total counts of *Enterobacteriaceae* family bacteria were on average 1.27–5.50 × 10^8^ CFU/g (the jejunum), 1.84–5.50 × 10^8^ CFU/g (the caecum) and 2.07–9.25 × 10^8^ CFU/g (the excreta) (Table S1). Our results are comparable to the study by Biernasiak et al., when the number of *Enterobacteriaceae* family bacteria in the excreta of chickens receiving probiotics was on average 10^8^ CFU/g [43].

The number of *Bifidobacterium* spp. and *Lactobacillus* spp. in samples of the intestinal content and the excreta of animals fed with OTA contaminated feed without additives decreased after 15 weeks of rearing (Table S1). The administration of feed supplemented with probiotics and synbiotics caused an increased count of these bacteria in the content of the jejunum, the caecum and the excreta of turkeys. In the case of new elaborated synbiotic preparations, the average numbers of *Bifidobacterium* spp. were 1.79–4.00 × 10^7^ CFU/g (the jejunum), 1.11–2.40 × 10^7^ CFU/g (the caecum) and 1.50–4.00 × 10^8^ CFU/g (the excreta) after 15 weeks of rearing. Furthermore, the average counts of *Lactobacillus* spp. in these groups of animals were 2.03–4.90 × 10^6^ CFU/g (the jejunum), 1.60–3.15 × 10^6^ CFU/g (the caecum) and 1.74–2.91 × 10^7^ CFU/g (the excreta) after 15 weeks of the experiment. It was found that the highest growth in number of these bacteria was in turkeys consuming feed supplemented with synbiotic C. In the content of intestines and the excreta, the numbers of *Bifidobacterium* spp. and *Lactobacillus* spp. in turkeys fed with feed supplemented with the synbiotic C were respectively 2 and 3 orders of magnitude higher compared to the control-positive group. Feeding turkeys with feed supplemented with probiotics resulted in an increase in the number of *Bifidobacterium* spp. and *Lactobacillus* spp. in the intestinal contents (1 order of magnitude) and the excreta of birds (2 and 1 order of magnitude, respectively) compared to control-positive group (Table S1).

In the jejunum content of control-positive turkeys, the count of *Clostridium* spp. and *Escherichia coli* increased to 1.20 × 10^8^ CFU/g and 2.20 × 10^8^ CFU/g, respectively, after 15 weeks of animals rearing (Table S1). In the case of turkeys consuming fed supplemented with synbiotics, the numbers of *Clostridium* spp. and *Escherichia coli* in the intestinal content after 6 weeks were significantly lower in comparison to control-positive group (2 and 1 orders of magnitude). The administration of turkeys with synbiotic supplemented feed for 15 weeks reduced the numbers of *Clostridium* spp. to 4.67 × 10^3^–1.67 × 10^4^ CFU/g (the jejunum), 1.05–2.77 × 10^5^ CFU/g (the caecum) and 2.25–8.85 × 10^4^ CFU/g (the jejunum). Moreover, the synbiotic supplementation of fodder resulted in a decrease in the numbers of *Escherichia coli* in the intestinal contents and the excreta of turkeys (3 and 4 orders of magnitude, respectively) compared to control-positive group. After 15 weeks of rearing, the numbers of *Escherichia coli* in these groups were 9.57 × 10^3^–2.47 × 10^4^ CFU/g (the jejunum), 4.00 × 10^4^–4.25 × 10^5^ CFU/g (the caecum) and 1.25–4.00 × 10^5^ CFU/g (the excreta). The best of results was found after the feed supplementation with the synbiotic C. Furthermore, probiotic preparations (BioPlus 2B and Cylactin) caused the reduction in the number *Clostridium* spp. and *Escherichia coli* by an average of 1–2 orders of magnitude compared to control-positive group (Table S1). Thus, all elaborated synbiotic preparations proved to be more effective in reducing the number of potentially pathogenic microorganisms in the intestinal content and the excreta of turkeys compared to tested commercial probiotic preparations.

The administration of probiotics and synbiotics to animals by 15 weeks did not have a statistically significant effect on the number of *Enterococcus* spp. and *Bacteroides* spp. in the intestinal content and the excreta of turkeys Big-6 (Table S1). In another study, no effect of two probiotics on the number of *Enterococcus* spp. in the intestinal microbiota of chickens was observed [44]. In previous studies in chickens regarding the developed synbiotic preparations, no change in the number of *Bacteroides* spp. as a result of treatment was observed either [25,26].

During the rearing of turkeys, the numbers of yeast in the intestinal content and the excreta of animals fed with synbiotics were significantly higher (the average of 2–3 orders the magnitude after 15 weeks) compared to birds consuming feed supplemented with probiotics or control-positive and control-negative animals (Table S1). Yeast are not commensal microorganisms in the intestinal of poultry. These results are very promising, because synbiotic preparations administrated to turkeys contained *Saccharomyces cerevisiae*. Hence, a high total count of yeast in samples obtained from these animals may indicate the survival of those microorganisms in the gastrointestinal tract of turkeys.

**Figure 1:**
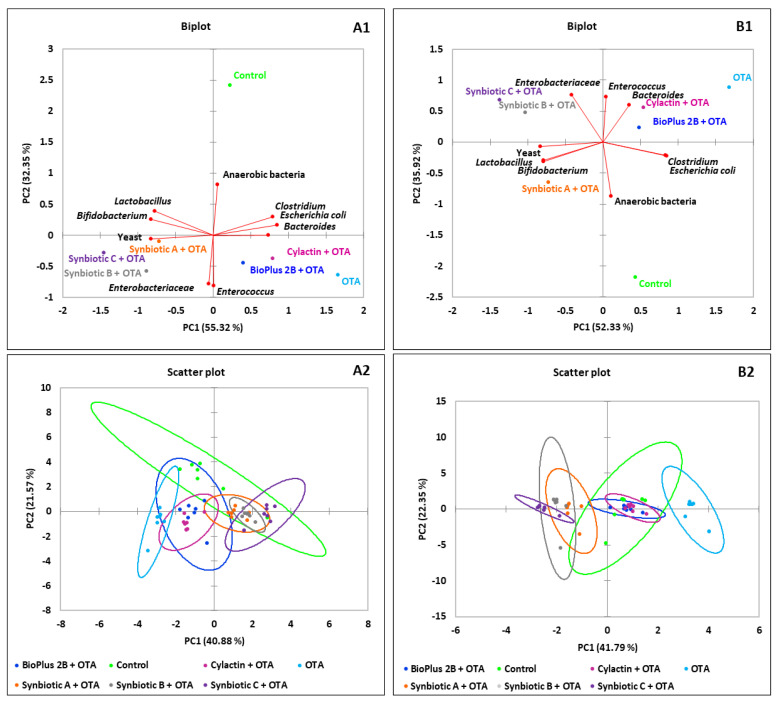

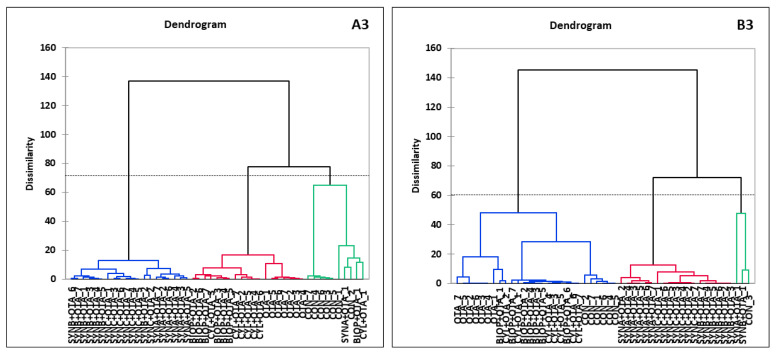
PCA plot of counts of microorganisms that dominate in the jejunum of turkeys. The scatter plot showing a clustering of individuals in groups of animals. AHC of 42 birds by the Ward’s method. The dotted line on the dendrogram is located at the node before the largest relative increase in dissimilarity level, (**A1**–**A3**) after 6 and (**B1**–**B3**) after 15 weeks of animals rearing.

**Figure 2:**
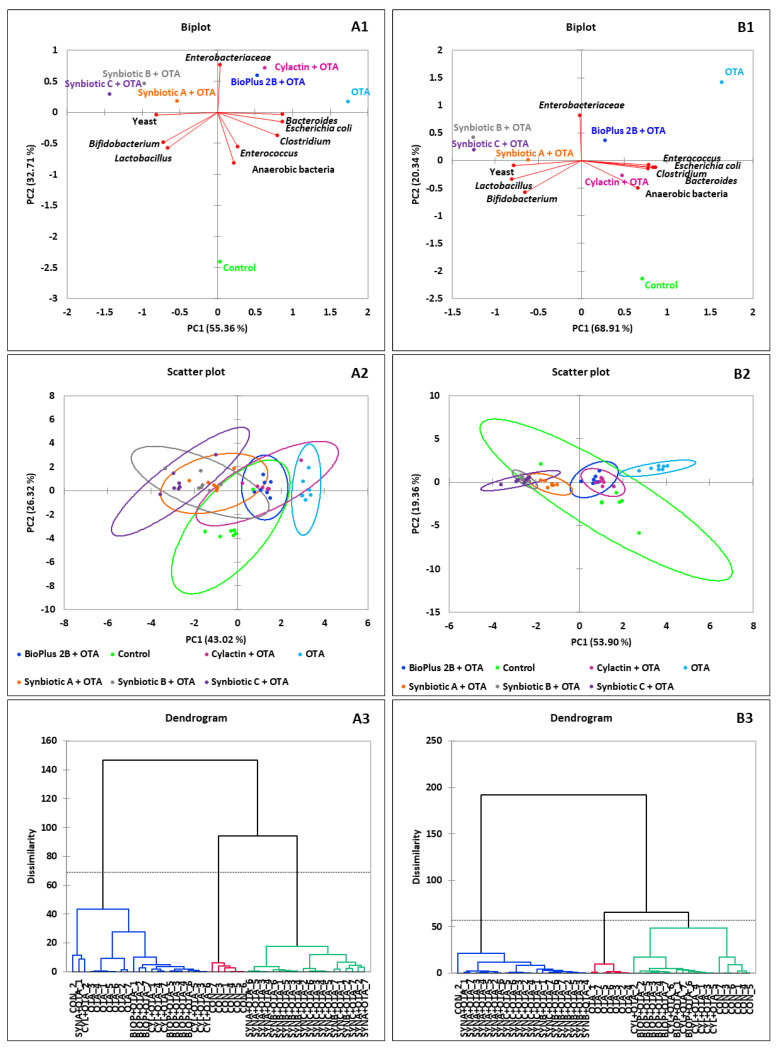
PCA plot of counts of microorganisms which dominate in the caecum of turkeys. The scatter plot showing a clustering of individuals in groups of animals. AHC of 42 birds by the Ward’s method. The dotted line on the dendrogram is located at the node before the largest relative increase in dissimilarity level; (**A1**–**A3**) after 6, and (**B1**–**B3**) after 15 weeks of animals rearing.

**Figure 3:**
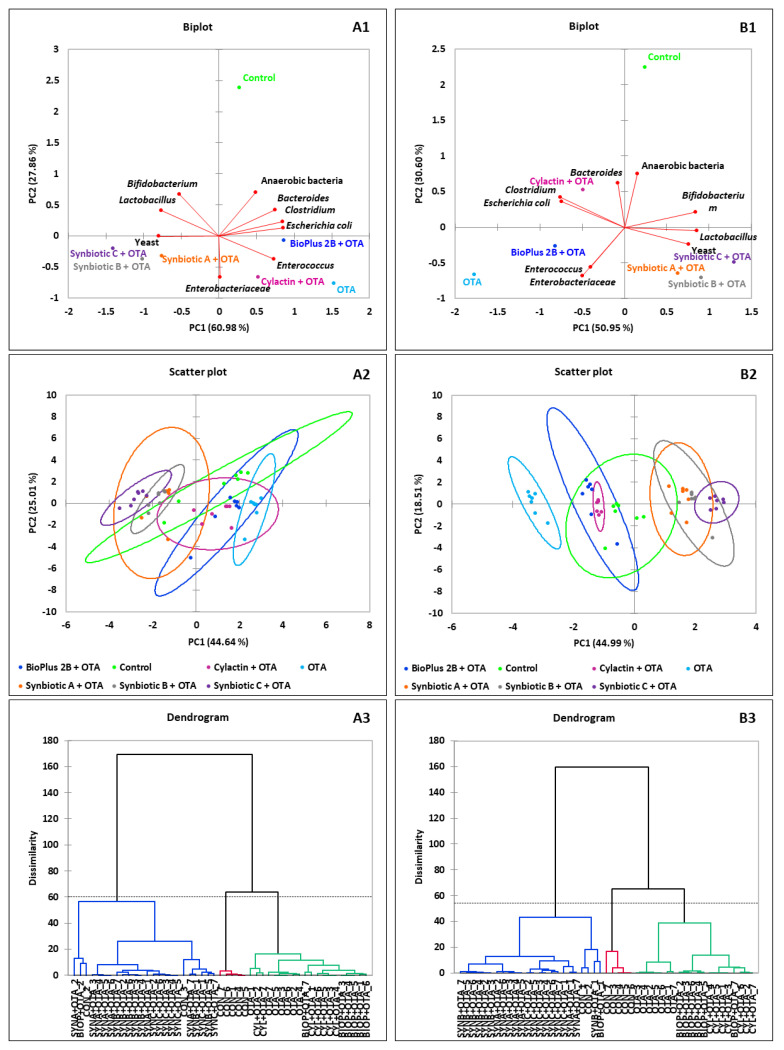

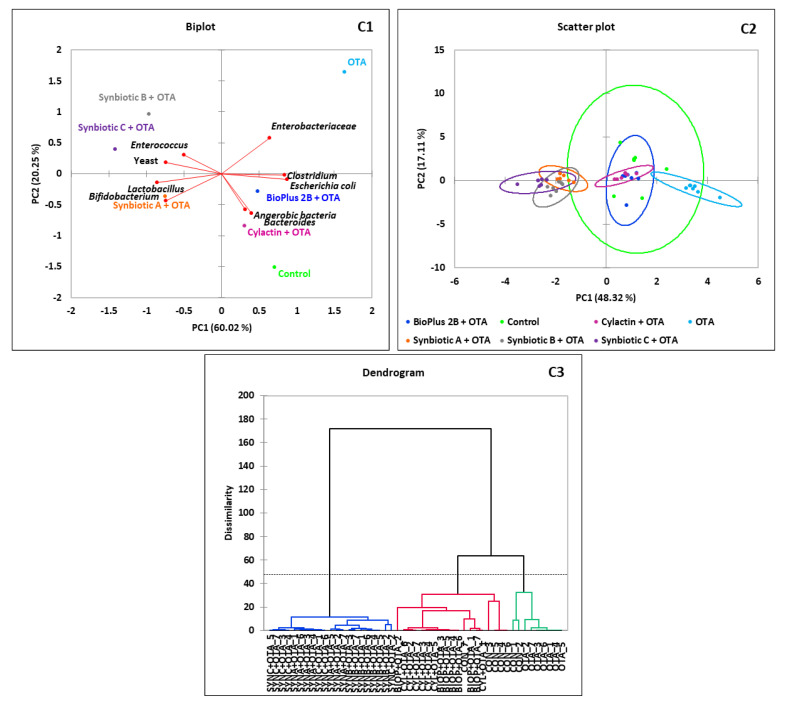
PCA plot of counts of microorganisms that dominate in the excreta of turkeys. The scatter plot showing a clustering of individuals in groups of animals in each time point of rearing, respectively. AHC of 42 birds by the Ward’s method. The dotted line on the dendrogram is located at the node before the largest relative increase in dissimilarity level, (**A1**–**A3**) after 3, (**B1**–**B3**) after 9 and (**C1**–**C3**) after 15 weeks of animals rearing.

In order to better visualize the results, principal component analysis (PCA) was used. The biplot for PC1 and PC2 showed the influence of tested synbiotic formulas and commercial preparations on dominant microorganisms in the jejunum content (Figure 1), the caecum content (Figure 2) and the excreta (Figure 3) in tested groups of animals. The visualization using the scatter plot showed a distinct clustering of individuals in each group of turkeys. In addition, from agglomerative hierarchical clustering analysis (AHC), it was determined that animals were divided into 3 clusters (Figure 1, Figure 2 and Figure 3). The clusters visible on presented dendrograms clearly show that the intestinal microbiota is an individual feature.

This study confirmed the beneficial effects of synbiotics A, B and C on the intestinal microbiota of turkeys. An important factor in these studies is the additional exposure of animals to contaminated OTA feed. Despite contamination of the feed, the synbiotic preparations had a very beneficial effect on the composition of the intestinal microbiota of animals and minimized the adverse effects of OTA. In addition, the effect of synbiotic preparations was better compared to commercial probiotic preparations BioPlus 2B and Cylactin.

### 2.4. The Effect of Synbiotics on the Activity of Fecal Enzymes in the Excreta of Turkeys

After 15 weeks of rearing, the lowest α-glucosidase activity was found in the excreta of turkeys fed with no additives—control-positive group (69.46 μMh/g) (Table 3). The activity of this enzyme was higher in the excreta of animal consuming feed with probiotics (BioPlus 2B and Cylactin) and was 75.91 μMh/g and 78.85 μMh/g, respectively. Furthermore, the highest α-glucosidase activity was found in groups of consuming feed supplemented with synbiotics A, B and C. Results obtained in these groups were 13–39% higher compared to the control-positive group and were 90.32 μMh/g, 78.77 μMh/g and 86.47 μMh/g, respectively. In the control-negative group, the activity of this enzyme after 15 weeks was 88.33 μMh/g (Table 3).

In the case of β-glucosidase, the highest activity of this enzyme was found in the excreta of control-positive animals and was on average 11.23 μMh/g after 15 weeks of treatment (Table 3). The administration of probiotics BioPlus 2B and Cylactin to animals reduced the activity of this enzyme to 8.31 μMh/g and 9.41 μMh/g, respectively. Furthermore, the average β-glucosidase activity in the excreta of animals consuming feed with the addition of synbiotics was on average 18–27% lower compared to the control-positive group and was, respectively, 8.50 μMh/g (Synbiotic A), 8.22 μMh/g (Synbiotic B) and 9.47 μMh/g (synbiotic C). In the control-negative group, the activity of this enzyme after 15 weeks was 8.09 μMh/g (Table 3).

After 15 weeks bird’s life, the average α-galactosidase activity in the excreta of turkeys consuming feed contaminated OTA was 81.03 μMh/g (Table 3). In the excreta of turkeys fed with fodder supplemented probiotics BioPlus 2B and Cylactin, the activity of this enzyme after 15 weeks was higher compared to control-positive group and was 87.17 μMh/g and 84.77 μMh/g, respectively. In turkeys fed with synbiotic supplemented feed, the highest α-galactosidase activity (9–17% higher compared to the control-positive group) was found, which was 93.84 μMh/g (synbiotic A), 96.85 μMh/g (synbiotic B) and 98.03 μMh/g (synbiotic C). The average α-galactosidase activity in the excreta of control turkeys was 94.00 μMh/g after 15 weeks of rearing (Table 3).

The administration of the OTA contaminated feed without supplements for 15 weeks increased the activity of β-galactosidase in the excreta of turkeys to an average of 97.77 μMh/g (Table 3). The addition of probiotic preparations to the feed caused a decrease in the activity of this enzyme to an average of 94.32 μMh/g (BioPlus 2B) and 93.59 μMh/g (Cylactin) after 15 weeks of turkeys rearing. The most effective turned out to be synbiotics B and C, which caused a decrease in the activity of β-galactosidase in the excreta of turkeys to 91.04 μMh/g and 87.08 μMh/g, respectively. In the control-negative group, the activity of this enzyme after 15 weeks was 89.33 μMh/g (Table 3).

The highest β-glucuronidase activity after 15 weeks of rearing was found in control-positive animals and averaged 51.92 μMh/g (Table 3). In the case of turkeys consuming feed with the addition of probiotics, the activity of this enzyme was reduced to 45.00 μMh/g (BioPlus 2B) and 49.42 μMh/g (Cylactin). Furthermore, the group of turkeys fed with synbiotic supplemented feed had the lowest β-glucuronidase activity in the excreta and was 50.50 μMh/g (synbiotic A), 47.99 μMh/g (synbiotic B) and 42.51 μMh/g (synbiotic C), respectively. The average β-glucuronidase activity in the excreta of control turkeys was 42.31 μMh/g after 15 weeks of rearing (Table 3). 

Some fecal enzymes (e.g., β-glucuronidase) can catalyze the release reaction of potentially harmful compounds, which can lead to carcinogenesis. Controlling the enzymatic activity of the intestinal microbiota by administering probiotics, prebiotics or synbiotics allows to reduce the activity of potentially harmful enzymes [45]. In the other research, it was found that 40-day lactic acid bacteria feed supplementation resulted in a decrease in β-glucuronidase and β-glucosidase activity in the excreta of broiler chickens [46]. In our own studies, administration of elaborated synbiotics also reduced the activity of these enzymes in the excreta of turkeys which additionally received the OTA contaminated feed. Shokryazdan et al. showed that the administration of a probiotic to broiler chickens significantly affects the activity of fecal enzymes [47]. The addition of three strains of *L. salivarius* to the feed resulted in a decrease in β-glucuronidase and β-glucosidase activity compared to control groups [47]. The activity of β-glucosidase and β-glucuronidase in 42-day-old chickens was reduced by 35% and 37% (dose 0.5 g/kg) and by 39% and 42% (dose 1.0 g/kg). Other studies tested the effects of Thepax^®^ probiotic (*S. cerevisiae*, *Lb. acidophilus*) and yogurt as a probiotic (*Lb. delbrueckii*, *Lb. thermophilus*) [48]. Broiler chickens received yogurt along with drinking water or feed with the addition of Thepax. Both supplements have significantly reduced the number of *E. coli* and *Clostridium perfringens*, which show high activity of potentially harmful enzymes. However, in the other studies, the effect of fructooligosaccharides (FOS) on the turkeys’ health was studied [49]. A diet containing 0.5%, 1% and 2% FOS resulted in reduction of the activity of β-glucuronidase and β-glucosidase. The research of Čokášová et al. studied the probiotic properties of *Lb. plantarum* and its effect on rats in which carcinogenesis has been chemically initiated [50]. In the feces of animals treated with the probiotic, an increased number of *Lactobacillus* spp. and a reduction in the number of coliforms were found. In addition, there was a decrease in β-glucuronidase and β-galactosidase activity and an increase in α-glucosidase and α-galactosidase activity. Śliżewska et al. investigated the effect of *Fusarium* mycotoxins, deoxynivalenol and zearalenone on β-glucosidase and β-glucuronidase activity in gilts [51]. After 6 weeks, a statistically significant increase in the activity of both enzymes was observed compared to the control group fed with uncontaminated feed. The reason for the increase in enzyme activity was the increase in the population of mesophilic microorganisms (e.g., *Escherichia coli*) due to the presence of mycotoxins in feed.

The results obtained in our study indicate a beneficial effect of the developed synbiotic preparations in the modulation of the intestinal microbiota and the activity of fecal enzymes in the excreta of turkeys fed with the OTA contaminated feed.

## 3. Conclusions

Results of this research showed that all elaborated synbiotic preparations have beneficial effect of on the turkey performance, the balance of the intestinal microbiota and the fecal enzymes activity in turkeys. Furthermore, the administration of synbiotics to animals fed OTA contaminated fodder produces a significant decrease in OTA concentration in the liver, the kidneys, the jejunum and the caecum of turkeys. All synbiotic formulas cause an increase in the count of beneficial intestinal microorganisms (*Lactobacillus* spp. and *Bifidobacterium* spp.) while a significant reduction in potentially pathogenic bacteria (*Escherichia coli* and *Clostridium* spp.) in the intestinal contents and the excreta of animals. Moreover, synbiotics produce beneficial changes in the fecal enzymes activity, causing a significant increase of α-glucosidase and α-galactosidase activity (beneficial enzymes) and a significant decrease of β-glucosidase, β-galactosidase and β-glucuronidase activity (potentially harmful enzymes) in comparison to the control group. Synbiotic C (the most complex preparation) proved to be the most effective one. The use of synbiotic preparations effectively minimizes the negative impact of the OTA contaminated feed on the turkey’s health. Hence, elaborated synbiotics can be successfully used as feed additives for turkeys for fattening.

## 4. Materials and Methods

### 4.1. Probiotic and Synbiotic Preparations

Synbiotic preparations (A, B and C) were elaborated in the Institute of Fermentation Technology and Microbiology of the Lodz University of Technology (Poland). The final formula of synbiotics was developed by the Department of Biotechnology and Food Microbiology of the Poznan University of Life Sciences. Each synbiotic preparation contained: 2.0 × 10^9^ CFU/g of *Lactobacillus* spp., 2.0 × 10^7^ CFU/g of *Saccharomyces cerevisiae* yeast and 2% inulin (prebiotic). The content of each microorganism in synbiotics A, B and C was equal. These probiotic strains were isolated from various sources (*Lb. paracasei* ŁOCK 1091—caecal content of sow; *Lb. pentosus* ŁOCK 1094—broiler chicken dung; *Lb. plantarum* ŁOCK 0860—plant silage; *Lb. reuteri* ŁOCK 1092—piglet caecal content; *Lb. rhamnosus* ŁOCK 1087—turkey dung and *S. cerevisiae* ŁOCK 0119—distillers’ yeast, grain) [26]. Commercial probiotic preparations contained 3.2 × 10^10^ CFU/g *Bacillus* spp. (BioPlus 2B; Biochem), and 1.0 × 10^10^ CFU/g *Enterococcus faecium* (Cylactin; DSM), respectively (Table 4). Probiotic microorganisms and prebiotic included in the synbiotic formulas have been tested for their probiotic and prebiotic properties according to the procedures recommended by FAO/WHO and EFSA [52,53]. Furthermore, the effectiveness of the developed synbiotic preparations in the feeding of monogastric animals has been confirmed so far in many in vitro and in vivo studies [16,25,26,54].

### 4.2. Animal Treatment

The study was conducted on 140 turkeys Big-6 (females), randomly divided into seven groups. Turkeys were housed in standard environmental conditions in separate cages (20 animals per cage) in one room of animal experimental laboratory. The animals were reared for 15 weeks from the first day of their life. The feed was natural contamination by the addition of OTA-infected wheat. The OTA concentration in feed ranged from 198.6 to 462.0 µg/kg depending on the type of feed (Table 5). For the examination period, during 15 weeks of life, in each group, birds were administrated synbiotic preparation (A, B or C) in a dose of 0.50 g/kg of feed or commercial probiotic preparation (BioPlus 2B or Cylactin) in a dose of 0.4 g/kg of feed ad libitum, respectively. The positive control was the group of birds that were administrated ad libitum the contaminated feed without additives. The negative control was the group of birds where OTA-free feed was administrated ad libitum without additives.

For the nutrition of animals, standard mixtures (nutritional value was adapted to the requirements of intensively growing birds) were used (Table 5). Mixtures were made in powder form. In their composition, there were cereal components (wheat), high-protein ingredients (post-extraction soya meal) and also soybean oil as an additional source of energy. In order to obtain the required level of exogenous amino acids, pure amino acids such as methionine, lysine and threonine were used. Mixtures contained mineral–vitamin premixes involving enzyme preparations (xylanase and phytase) and coccidiostat Clinacox (Starter 1,2 and Grower 1). Detailed parameters of the applied feed are presented in Table 5.

**Figure 4:**
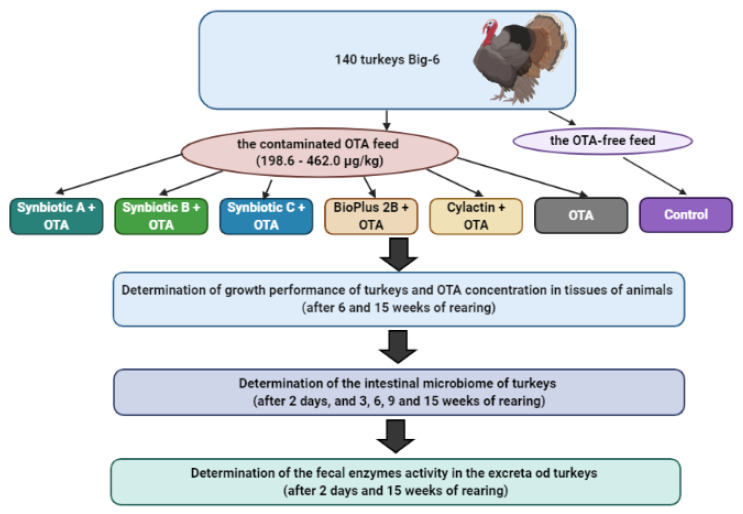
The scheme of the experiment.

Birds were grown in the Department of Animal Nutrition and Fodder Knowledge, University of Warmia and Mazury (Olsztyn, Poland). Experiments were conducted after obtaining the approval of the Local Ethical Commission for Animal Testing in Olsztyn (Poland) according to resolution No. 14/2012 (from 29 February 2012). All studies were performed in accordance with relevant guidelines and regulations. The scheme of the conducted experiment is presented in Figure 4.

### 4.3. Determination of the Turkey Performance

The turkeys’ rearing time was 15 weeks. Turkeys were observed daily in order to detect potential undesirable effects or deaths of birds. The body weight of animals in individual study groups was determined after 6 and 15 weeks of life. Final production parameters such as daily cumulative mortality rate (%), feed conversion ratio (FCR), and European Production Efficiency Factor (EPEF) were determined according to the formulas [16]:

$$\begin{array}{c} \mathrm{daily}\;\mathrm{cumulative}\;\mathrm{mortality}\;\mathrm{rate}\;\left(\%\right)=\;\frac{\mathrm{the}\;\mathrm{number}\;\mathrm{of}\;\mathrm{death}\;\mathrm{turkeys}\;\left(\mathrm{pc}.\right)\times 100\%}{\mathrm{the}\;\mathrm{number}\;\mathrm{of}\;\mathrm{turkeys}\;\mathrm{in}\;\mathrm{research}\;\left(\mathrm{pc}.\right)}\;\\ \mathrm{FCR}=\frac{\mathrm{the}\;\mathrm{feed}\;\mathrm{consumption}\;\left(\mathrm{kg}\right)}{\mathrm{the}\;\mathrm{body}\;\mathrm{weight}\;\mathrm{gain}\;\left(\mathrm{kg}\right)}\\ \mathrm{EPEF}=\frac{\mathrm{the}\;\mathrm{liveability}\;\left(\%\right)\times \mathrm{the}\;\mathrm{body}\;\mathrm{weight}\;\left(\mathrm{kg}\right)\times 100}{\mathrm{the}\;\mathrm{age}\;\left(\mathrm{days}\right)\times \mathrm{the}\;\mathrm{feed}\;\mathrm{conversion}\;\mathrm{ratio}\;\left(\mathrm{kg}\right)} \end{array}$$

### 4.4. Determination of the Ochratoxin A Content in Feed, Tissues and the Intestinal Content

The concentrations of OTA in diets, wheat grain, tissue and intestine content were determined by immunoaffinity column clean-up and HPLC-fluorescence detection in accordance with Polish Standard PN-EN 16007-2012-U [55]. Deep-frozen tissue samples and intestinal contents were lyophilized at −70 °C using a lyophilizer (Alpha 1–2 LDplus, CHRIST, Germany). The extracts concentrations were measured by high-performance liquid chromatography (HPLC) (LC–20AD, Shimadzu, Japan). The HPLC apparatus consisted of Jupiter 5u C18 300A column chromatography (Phenomenex) 250 × 4.60 mm, column operating temperature: 24 °C; mobile phase—acetonitrile/methanol/aqueous acetic acid (35; 35; 30 v/v/v); flow rate: 0.5 mL/min; dosing volume: 100 μL; fluorescence detector: λex = 333 nm, λem = 467 nm. The qualitative interpretation of the obtained chromatograms was carried out by comparing the retention time of the analyzed toxin in the appropriate standard solution with the retention time of a given analyte in the actual sample. Quantitative analysis was carried out by reading the content of mycotoxin in the sample from the standard curve and making appropriate calculations. Each sample was analyzed in two parallel repetitions.

### 4.5. Determination of Intestinal Microbiota of Turkeys

The composition of dominant microorganisms in turkeys was determined after 3, 9 and 15 weeks (the excreta), after 6 and 15 weeks (the jejunum as part of the small intestine and the caecum) of rearing in seven randomly selected turkeys (three repetitions) in each experimental group. The count of analyzed microorganisms was determined using the culture method in accordance with the PN-ISO standards in triplicate, using selective microbial media [39]. The total anaerobic bacterial count (PCA, Merck), *Enterobacteriaceae* family bacteria count (VRBD, Merck), *Escherichia coli* count (TBX, Merck) and the count of bacteria belonging to the genes *Lactobacillus* (MRS, Merck), *Bifidobacterium* (RCA), *Clostridium* (TSC with D-cycloserine, Merck), *Enterococcus* (BAA, Merck), *Bacteroides* (VL, Merck) were determined. Considering the presence of yeast in the composition of synbiotic preparations, the yeast count was also determined on SDA (Merck). Plates were incubated in conditions appropriate for a given group of microorganisms: unlimited oxygen at 37 °C for 48 h (*Lactobacillus*, *Enterococcus*, *Enterobacteriaceae*), 44 °C for 48 h (*Escherichia coli*), 30 °C for five days (total yeast count) and in limited oxygen at 37 °C for 48 h (total anaerobic count, *Bifidobacterium*, *Clostridium* and *Bacteroides*) [25].

### 4.6. Determination of the Activity of Fecal Enzymes in the Excreta of Turkeys

The fecal enzymes activity in the excreta of turkeys was determined after 2 days and 15 weeks of the animals rearing in seven randomly selected turkeys (three repetitions) in each experimental group. The activity of α-glucosidase, β-glucosidase, α-galactosidase, β-galactosidase and β-glucuronidase in the excreta of turkeys was determined using the spectrophotometric method using the multi-plate microplate reader TriStar2 S LB 942 (Berthold Technologies, Germany). In order to prepare samples for analysis, an ultrasonic disintegrator (Sonificator Cole-Parmer Instrument Co., USA) was used with appropriate parameters (amplitude 60 Hz, pulse 6 s, break 2 s, total time 5 min). The method is based on a color reaction between the appropriate substrate and the determined enzyme. Absorbance was measured at λ = 400 nm (α-glucosidase, α-galactosidase, β-galactosidase), λ = 450 nm (β-glucosidase) and λ = 540 nm (β-glucuronidase). The activity of the determined enzyme [μMh/g] was expressed as the amount of p-nitrophenol [μM] (α-glucosidase, β-glucosidase, α-galactosidase, β-galactosidase) or phenolphthalein (β-glucuronidase) released under specific conditions within 1 h on 1 g of the excreta.

## 5. Statistical Analysis

The limit of quantification (the LOQ) for the OTA content in feed, tissues and the intestinal content was 0.025–0.03 µg/kg. The normality of the distribution of variables was examined with Shapiro–Wilk’s test, and the homogeneity of variances was tested with Bartlett’s test [16]. Following the confirmation of normality and equal variance, results were analyzed using analysis of variance with a one-way ANOVA test and Tukey’s post hoc test. Differences between samples with normal distribution were also evaluated by Student’s t-test. In addition, Principal components’ analysis (PCA) and Agglomerative hierarchical cluster analysis (AHC) of overall diversity in the intestinal microbiota was performed to compare all groups of turkeys at the time of probiotics and synbiotics supplementation. Statistical analysis was performed using XLSTAT Software (Addinsoft, SARL, Paris, France) at the significance level of *p* < 0.05. The results were presented as mean ± standard deviation (SD).

## 6. Patents

The strains from the new elaborated synbiotic preparations possess full probiotic documentation described in patent applications and the patent description [56,57,58,59,60,61].

## Figures and Tables

**Table 1:** Rearing parameters of turkeys after 6 and 15 weeks of life. One-way ANOVA with post-hoc Tukey’s test (^a,b^—values in rows with different letters are significantly different for *p* < 0.05).

The Age of Birds (Weeks)	Feed Additives	The Body Weight (Mean ± SD) (kg)	Daily Cumulative Mortality Rate (%)	FCR	EPEF
6	Control	1.85 ± 0.07	0.83	1.76 ^b^	248.18 ^a^
	OTA	1.65 ± 0.17	0.24	2.31 ^a^	169.66 ^b^
	Synbiotic A + OTA	1.78 ± 0.15	0.24	2.23 ^a^	189.60 ^ab^
	Synbiotic B + OTA	1.67 ± 0.14	0.24	2.34 ^a^	169.52 ^b^
	Synbiotic C + OTA	1.78 ± 0.15	0.36	2.32 ^a^	182.02 ^ab^
	BioPlus 2B + OTA	1.64 ± 0.19	0.24	2.36 ^a^	165.06 ^b^
	Cylactin + OTA	1.65 ± 0.16	0.36	2.37 ^a^	165.17 ^b^
15	Control	9.67 ± 0.13 ^a^	0.83	2.32	393.65 ^a^
	OTA	7.60 ± 0,40 ^b^	0.48	2.79	257.27 ^b^
	Synbiotic A + OTA	7.80 ± 0.47 ^b^	0.24	2.71	273.46 ^b^
	Synbiotic B + OTA	7.63 ± 0.48 ^b^	0.24	2.80	258.91 ^b^
	Synbiotic C + OTA	7.72 ± 0.67 ^b^	0.36	2.80	261.65 ^b^
	BioPlus 2B + OTA	7.63 ± 0.51 ^b^	0.24	2.79	258.91 ^b^
	Cylactin + OTA	7.79 ± 0.52 ^b^	0.36	2.80	264.97 ^b^

**Table 2:** The content of OTA in the liver, the kidneys, and the jejunum and the caecum content of turkeys after 6 and 15 weeks of life. One-way ANOVA with post-hoc Tukey’s test (^a,b,c,d^—values in rows with different letters are significantly different for *p* < 0.05).

The Age of Birds (weeks)	Feed Additives	The Content of OTA (Mean ± SD) (µg/kg)			
		The Liver	The Kidneys	The Jejunum	The Caecum
6	Control	0.00 ± 0.00	0.00 ± 0.00 ^d^	0.00 ± 0.00 ^d^	0.00 ± 0.00 ^d^
	OTA	4.51 ± 1.55	5.78 ± 0.61 ^a^	151.82 ± 46.88 ^a^	100.36 ± 4.85 ^a^
	Synbiotic A + OTA	3.40 ± 0.27	2.93 ± 0.52 ^c^	57.04 ± 18.87 ^c^	78.79 ± 7.49 ^b^
	Synbiotic B + OTA	2.46 ± 0.61	3.62 ± 0.56 ^bc^	105.97 ± 18.31 ^b^	46.87 ± 1.73 ^c^
	Synbiotic C + OTA	2.95 ± 0.36	3.66 ± 1.50 ^bc^	106.83 ± 15.22 ^b^	111.82 ± 11.11 ^a^
	BioPlus 2B + OTA	3.60 ± 0.96	4.46 ± 1.02 ^ab^	102.81 ± 16.83 ^b^	53.73 ± 1.84 ^c^
	Cylactin + OTA	3.52 ± 0.41	3.67 ± 1.11 ^bc^	126.07 ± 29.28 ^ab^	106.43 ± 7.65 ^a^
15	Control	0.00 ± 0.00 ^c^	0.00 ± 0.00 ^b^	0.00 ± 0.00 ^d^	0.00 ± 0.00 ^c^
	OTA	3.80 ± 0.68 ^a^	4.54 ± 0.74 ^a^	177.62 ± 33.04 ^a^	73.34 ± 50.21 ^a^
	Synbiotic A + OTA	2.16 ± 0.24 ^b^	3.17 ± 0.73 ^a^	97.40 ± 33.36 ^bc^	34.63 ± 7.37 ^bc^
	Synbiotic B + OTA	2.14 ± 0.28 ^b^	3.23 ± 1.24 ^a^	96.21 ± 43.98 ^c^	54.93 ± 2.16 ^ab^
	Synbiotic C + OTA	2.74 ± 0.42 ^b^	3.52 ± 0.54 ^a^	147.26 ± 24.38 ^ab^	45.98 ± 3.92 ^ab^
	BioPlus 2B + OTA	2.36 ± 0.57 ^b^	3.82 ± 1.77 ^a^	163.31 ± 19.80 ^a^	60.16 ± 34.54 ^ab^
	Cylactin + OTA	2.83 ± 1.18 ^b^	4.50 ± 0.59 ^a^	116.66 ± 33.23 ^bc^	40.26 ± 11.43 ^ab^

**Table 3:** The activity of fecal enzymes in excreta of turkeys after 2 days and 15 weeks of rearing. One-way ANOVA with post-hoc Tukey’s test (^a,b,c,d^ values in rows with different letters are significantly different for *p* < 0.05).

Age	Feed Additives	The Activity of Enzyme (Mean ± SD) [μMh/g]				
		α-glucosidase	β-glucosidase	α-galactosidase	β-galactosidase	β-glucuronidase
2 days	Control	86.37 ± 1.32	7.01 ± 1.51	81.55 ± 2.88	65.36 ± 2.07	39.05 ± 2.14
15 weeks	Control	88.33 ± 2.77 ^a^	8.09 ± 1.97 ^b^	94.00 ± 2.89 ^a^	89.33 ± 2.38 ^cd^	42.31 ± 2.10 ^c^
	OTA	69.46 ± 0.35 ^c^	11.23 ± 1.14 ^a^	81.03 ± 1.89 ^c^	97.77 ± 1.57 ^a^	51.92 ± 2.53 ^a^
	Synbiotic A + OTA	90.32 ± 1.66 ^a^	8.50 ± 1.04 ^b^	93.84 ± 0.98 ^a^	94.11 ± 1.52 ^b^	50.50 ± 1.69 ^a^
	Synbiotic B + OTA	78.77 ± 4.55 ^b^	8.22 ± 1.11 ^b^	96.85 ± 3.59 ^a^	91.04 ± 1.04 ^c^	47.99 ± 2.90 ^ab^
	Synbiotic C + OTA	86.47 ± 2.48 ^a^	9.47 ± 1.14 ^ab^	98.03 ± 3.47 ^a^	87.08 ± 0.89 ^d^	42.51 ± 1.00 ^c^
	BioPlus 2B + OTA	75.91 ± 2.99 ^bc^	8.31 ± 0.67 ^b^	87.17 ± 1.80 ^b^	94.32 ± 0.87 ^b^	45.00 ± 2.36 ^bc^
	Cylactin + OTA	78.85 ± 2.86 ^b^	9.41 ± 0.67 ^ab^	84.77 ± 1.88 ^a^	93.59 ± 1.67 ^b^	49.42 ± 2.17 ^ab^

**Table 4:** The composition of new elaborated synbiotic formulas and commercial probiotic preparations.

Type of Preparation	Name of Preparation	Probiotic Microorganisms	Prebiotics
**Synbiotics**	A	*Lb. plantarum* ŁOCK 0860 *Lb. reuteri* ŁOCK 1092 *Lb. pentosus* ŁOCK 1094 *S. cerevisiae* ŁOCK 0119	inulin
	B	*Lb. plantarum* ŁOCK 0860 *Lb. reuteri* ŁOCK 1092 *Lb. pentosus* ŁOCK 1094 *Lb. rhamnosus* ŁOCK 1087 *S. cerevisiae* ŁOCK 0119	
	C	*Lb. plantarum* ŁOCK 0860 *Lb. reuteri* ŁOCK 1092 *Lb. pentosus* ŁOCK 1094 *Lb. rhamnosus* ŁOCK 1087 *Lb. paracasei* ŁOCK 1091 *S. cerevisiae* ŁOCK 0119	
**Probiotics**	BioPlus 2B	*B*. *licheniformis* DSM 5749 *B*. *subtilis* DSM 5750	without
	Cylactin	*E*. *faecium* NCIMB 10415 (SF68)	

**Table 5:** The composition and nutritional value of turkey compound feeds.

	Type of Feed	Starter 1 0–3 Weeks	Starter 2 4–6 Weeks	Grower 1 7–9 Weeks	Grower 2 10–12 Weeks	Finisher 13–15 Weeks
The Composition of Feed (g/kg)						
Wheat		261.9	310.3	416.7	515.8	590.4
Corn		200.0	200.0	150.0	100.0	100.0
Soybean meal		358.2	360.9	347.4	300.0	225.1
Full-fat soybeans		100.0	50.0	-	-	-
Blood meal		20.0	10.0	-	-	-
Soybean oil		5.2	19.2	39.1	45.1	47.8
L-lysine HCl		3.1	3.6	3.7	3.2	4.0
DL-methionine		3.5	2.6	2.4	2.6	2.5
L-threonine		0.7	0.7	1.1	0.7	1.0
Limestone		18.8	14.5	13.4	11.1	9.7
Monocalcium phosphate		22.1	19.9	17.7	13.1	11.1
Sodium bicarbonate		0.1	1.3	1.2	0.7	0.7
NaCl		2.0	1.9	2.2	2.6	2.7
Feed enzymes		0.1	0.1	0.1	0.1	0.1
Premix *		5.0	5.0	5.0	5.0	5.0
The nutritional value of feed						
Metabolizable energy (kcal/kg)		2800	2880	3000	3100	3200
Crude protein (g)		27.00	25.20	23.00	21.50	19.00
Lysine (%)		1.77	1.65	1.45	1.30	1.17
Methionine and Cysteine (%)		1.15	1.02	0.95	0.93	0.85
Ca (g)		1.40	1.20	1.15	1.00	0.90
Available P (g)		0.70	0.65	0.60	0.50	0.45
Na (g)		0.13	0.15	0.15	0.15	0.15
The mycotoxin content in feed						
Ochratoxin A (µg/kg) **		198.6	251.6	331.0	397.2	462.0

* The composition of the premix for: Starter—12 500 IU Vitamin A, 4 500 IU Vitamin D_3_, 87.5 mg Vitamin E, 3.75 mg Vitamin K_3_, 3.5 mg Vitamin B_1_, 10 mg Vitamin B_2_, 75 mg Niacin, 22.5 mg Pantothenic acid, 6.0 mg Vitamin B_6_, 30 µg Vitamin B_12,_ 2.5 mg Folic acid, 400 µg Biotin, 800 mg Choline Chloride, 92.5 mg Fe, 130 mg Mn, 20 mg Cu, 105 mg Zn, 2.5 mg J, 0.3 mg Se; Grower—11 500 IU Vitamin A, 4 140 IU Vitamin D_3_, 80.5 mg Vitamin E, 3.45 mg Vitamin K_3_, 3.22 mg Vitamin B_1_, 9.2 mg Vitamin B_2_, 69 mg Niacin, 20.7 mg Pantothenic acid, 5.52 mg Vitamin B_6_, 37.6 µg Vitamin B_12,_ 2.3 mg Folid Acid, 368 µg Biotin, 600 mg Choline Chloride, 85.1 mg Fe, 120 mg Mn, 18.4 mg Cu, 96.6 mg Zn, 2.3 mg J, 0.26 mg Se; Finisher—10 500 IU Vitamin A, 3 780 IU Vitamin D_3_, 66.5 mg Vitamin E, 2.85 mg Vitamin K_3_, 2.66 mg Vitamin B_1_, 7.6 mg Vitamin B_2_, 57 mg Niacin, 17.1 mg Pantothenic acid, 4.6 mg Vitamin B_6_, 22.8 µg Vitamin B_12,_ 1.9 mg Folid Acid, 304 µg Biotin, 400 mg Choline Chloride, 70.3 mg Fe, 98.8 mg Mn, 15.2 mg Cu, 79.8 mg Zn, 1.9 mg J, 0.23 mg Se. ** It does not apply to the negative control group.

## Supplementary Materials

The following are available online at https://www.mdpi.com/2072-6651/12/9/578/s1. Table S1: Counts of microorganisms which dominate in the content of the jejunum and the caecum and also the excreta of turkeys. One-way ANOVA with post-hoc Tukey’s test (*p* < 0.05).
